# Identification of Inflammation-Related Biomarker Lp-PLA2 for Patients With COPD by Comprehensive Analysis

**DOI:** 10.3389/fimmu.2021.670971

**Published:** 2021-05-21

**Authors:** Mingming Deng, Yan Yin, Qin Zhang, Xiaoming Zhou, Gang Hou

**Affiliations:** ^1^ Department of Pulmonary and Critical Care Medicine, Center of Respiratory Medicine, China-Japan Friendship Hospital, Beijing, China; ^2^ Graduate School of Peking Union Medical College, Chinese Academy of Medical Sciences, Peking Union Medical College, Beijing, China; ^3^ National Center for Respiratory Medicine, Beijing, China; ^4^ Institute of Respiratory Medicine, Chinese Academy of Medical Sciences, Beijing, China; ^5^ National Clinical Research Center for Respiratory Diseases, Beijing, China; ^6^ Department of Pulmonary and Critical Care Medicine, First Hospital of China Medical University, Shenyang, China; ^7^ Department of Pulmonary and Critical Care Medicine, Fourth Hospital of China Medical University, Shenyang, China

**Keywords:** COPD, biomarker, Lp-PLA2, PLA2G7, exercise tolerance

## Abstract

**Purpose:**

Chronic obstructive pulmonary disease (COPD) is a complex and persistent lung disease and lack of biomarkers. The aim of this study is to screen and verify effective biomarkers for medical practice.

**Methods:**

Differential expressed genes analysis and weighted co-expression network analysis were used to explore potential biomarker. Gene Ontology (GO) enrichment, Kyoto Encyclopedia of Genes and Genomes (KEGG) analysis and Gene set enrichment analysis (GSEA) analysis were used to explore potential mechanism. CIBERSORTx website was used to evaluate tissue-infiltrating immune cells. Enzyme-linked immunosorbent assay (ELISA) was used to assess the concentrations of the Lp-PLA2 in serum.

**Results:**

Ten genes were selected *via* combined DEGs and WGCNA. Furthermore, PLA2G7 was choose based on validation from independent datasets. Immune infiltrate and enrichment analysis suggest PLA2G7 may regulate immune pathway *via* macrophages. Next, Lp-PLA2(coded by PLA2G7 gene) level was upregulated in COPD patients, increased along with The Global Average of COPD (GOLD) stage. In additional, Lp-PLA2 level was significant correlate with FEV1/FVC, BMI, FFMI, CAT score, mMRC score and 6MWD of COPD patients. Finally, the predictive efficiency of Lp-PLA2 level (AUC:0.796) and derived nomogram model (AUC:0.884) in exercise tolerance was notably superior to that of the sit-to-stand test and traditional clinical features.

**Conclusion:**

Lp-PLA2 is a promising biomarker for COPD patients and is suitable for assessing exercise tolerance in clinical practice.

## Introduction

Chronic obstructive pulmonary disease (COPD) is a complex and persistent lung disease, kills more than 3 million people worldwide every year (1). Despite progress in comprehensive therapies and prevention of acute exacerbations over past decades, therapeutic advances in ameliorate disease progression or reduce mortality remains few (2, 3). Therefore, it is needed to have a better understanding of the complex disease mechanisms and explore better biomarkers to assess and monitor disease risk and prognosis.

Impaired exercise tolerance is one of the clinical features of COPD, leading disease progression and increase mortality of patients (4). Therefore, it is needed to assess and monitor exercise tolerance effectively. The 6-minute walk test (6MWT) is a reliable and widely used measure of exercise capacity (5). 6MWT need a suitable venue and professional training. However, it has been difficult to popularize in primary medical institutions due to need for suitable venue and professional training. It is important to establish a screening method that is highly accurate, simple, and can be performed in medical facility.

Recent years, transcriptomics research has shown good application prospects in helping researchers deepen their understanding of disease mechanisms and efficiently explore biomarkers (6–8). Several COPD studies (9–11) have identified hundreds of biomarkers *via* differentially analysis based on gene expression profiles from public databases. However, these biomarkers lack sufficiently sensitivity and the specificity due to samples heterogeneity and many confounding factors. Therefore, future biomarker studies need comprehensive and insight analysis instead of simplistic differential analysis between COPD patients and control.

In this study, we screened out the hub gene PLA2G7 *via* combined differentially expressed gene (DEG) analysis, weighted co-expression network analysis (WGCNA), and validation in a separate dataset. The level of Lp-PLA2 (encoded by the PLA2G7 gene) was assessed as a potential biomarker to predict poor exercise tolerance in COPD patients in clinical practice.

## Material and Methods

### Dataset Preparation

Microarray datasets were screened from Gene Expression Omnibus (GEO, http://www.ncbi.nlm.nih.gov/geo). The selection criteria were as follows: 1. Lung tissue samples from COPD patients and normal smokers’ lungs were included; lung cancer samples were excluded; 2. COPD patients had pulmonary function test data; and 3. datasets should contain at least 20 COPD patients and tissue samples from smokers. Based on these criteria, the GSE76925 and GSE38974 datasets were obtained. GSE76925 contained 111 lung tissue samples from COPD patients and 40 lung tissue samples from normal smokers. GSE38974 contained 26 lung tissue samples from COPD patients and nine lung tissue samples from normal smokers. GSE76925 was used to screen DEGs and for WGCNA. GSE38974 was used for validation of hub genes. GSE69818 was used to analysis the relationship between the expression level of hub genes and clinical feature. GSE69818 contained 70 former smokers with COPD (11 patients with The Global Average of COPD (GOLD) stage 1, 41 patients with GOLD stage 2, 9 patients with GOLD stage 3, and 9 patients with GOLD stage 4).

### Differentially Expressed Genes and Enrichment Analysis

The R package “Limma” was used to identify DEGs between COPD samples and normal smoker samples. An adjusted P-value <0.05 and |log_2_fold change| ≥1 was used as cut-off values. The R package “clusterProfiler” was used for Gene Ontology (GO) enrichment and Kyoto Encyclopedia of Genes and Genomes (KEGG) analysis. P <0.05 was considered statistically significant.

### Gene Set Enrichment Analysis

Gene set enrichment analysis (GSEA) was performed as previously described (12). The MSigDB KEGG gene set was used as a reference.

### Evaluation of Tissue-Infiltrating Immune Cells

In this study, we used the “CIBERSORTx” website to estimate the fraction of 22 types of immune cells among GSE76925 samples. CIBERSORTx (13) is an analytical tool to impute gene expression profiles and provide an estimate of the abundance of member cell types in a mixed cell population using gene expression data.

### Weighted Co-Expression Network Analysis

A total of 4283 genes (according to variance) were extracted for WGCNA using a “WGCNA” package. The adjacency matrix was converted into the topological overlap matrix (TOM) when the power of β was equal to 3 (R^2^ = 0.906). Similar modules were merged following a height cutoff of 0.25. The module showing the highest correlation with clinical features was selected to explore its biological function through GO and KEGG analyses.

### Patients and Clinical Information

The study included 92 stable-stage COPD patients, 16 healthy smokers and 10 never-smokers recruited from the Department of Respiratory and Critical Care Medicine of the First Hospital of China Medical University. Clinical features including age, sex, height, weight, pulmonary function, and mMRC (modified British Medical Research Council) and COPD Assessment Test (CAT) results were obtained from medical records. Five-repetition sit-to-stand test (5STS), the 30-second sit-to-stand test (30STS), and the 6MWT were performed as described previously (14).

### Enzyme-Linked Immunosorbent Assay (ELISA)

Lp-PLA2 levels were determined using the human Lp-PLA2 enzyme immunoassay kit (CSB-E08319h, CUSABIO, Wuhan, China) according to the manufacturer’s instructions.

### Statistical Analysis

Statistical analyses were performed using SPSS 13.0 software (IBM, Armonk, NY, USA). The association between continuous variables was assessed using Spearman’s correlation coefficient. Relationships between categorical variables were analyzed using the chi-square test. For continuous variables, differences between three or more groups were assessed using one-way analysis of variance (ANOVA) with the post-hoc Tukey multiple comparison test (for normally distributed data) or Kruskal-Wallis test (for non-normal distribution). Differences between two groups were assessed using the *t*-test (normally distributed data) or Mann-Whitney test (non-normal distribution). P values <0.05 were considered statistically significant.

## Results

### Identification of DEGs and Enrichment Analysis in Samples From COPD Patients and Normal Smokers

**Figure 1:**
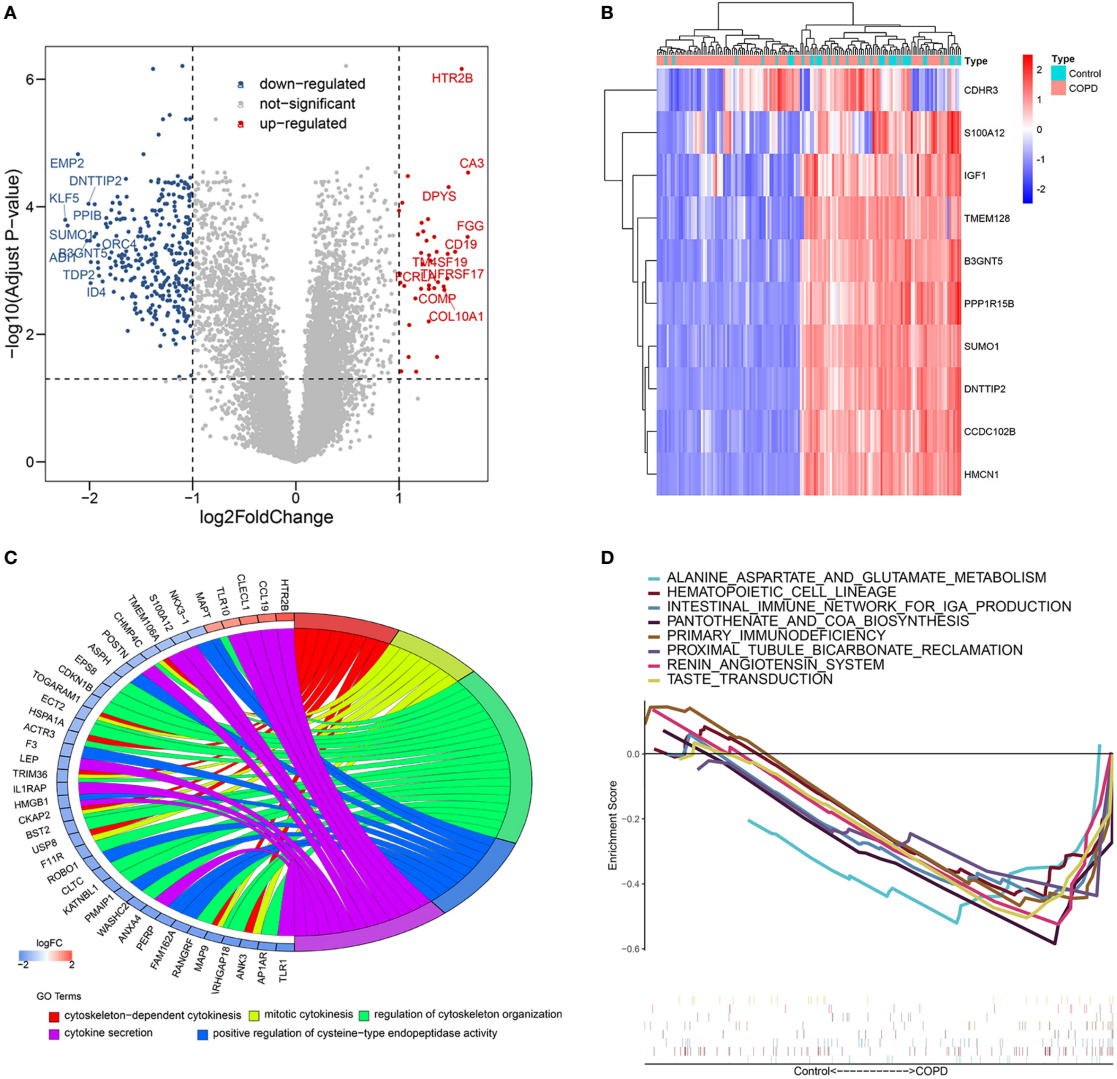
Differential expressed genes analysis and Volcano map **(A)** and heatmap **(B)** of differential expressed genes; **(C)** Chord plot depicting the relationship between genes and Gene ontology (GO) terms of biological process; **(D)** GSEA showed eight pathways enriched in COPD patients.

DEGs in lung tissue samples from COPD patients and normal smokers were analyzed using the “Limma” package. As shown in **Figures 1A**, **B**, 39 significantly upregulated genes and 313 significantly downregulated genes were identified. GO analysis of the 352 DEGs showed that genes were mainly involved in biological processes (BP) associated with the cytoskeleton and cytokines (**Figure 1C** and **Table S1**). The results of GSEA analysis showed that VEGF_SIGNALING_PATHWAY, NUCLEOTIDE_EXCISION_REPAIR, and LONG_TERM_DEPRESSION pathways were enriched in the normal smokers group compared with COPD (**Table S2**). The results of GSEA analysis showed that “ALANINE_ASPARTATE_AND_GLUTAMATE_METABOLISM”, “HEMATOPOIETIC_CELL_LINEAGE”, “INTESTINAL_IMMUNE_NETWORK_FOR_IGA_PRODUCTION”, “KEGG_PANTOTHENATE_AND_COA_BIOSYNTHESIS”, “PRIMARY_IMMUNODEFICIENCY”, “PROXIMAL_TUBULE_BICARBONATE_RECLAMATION”, “RENIN_ANGIOTENSIN_SYSTEM”, and “TASTE_TRANSDUCTION” pathways were enriched in the COPD group compared with normal smokers (**Figure 1D** and **Table S3**). Taken together, these results identify potential biomarkers and abnormal signaling pathways involved in the progression of COPD.

### Immune Landscape Associated With the Characteristics of COPD Patients

**Figure 2:**
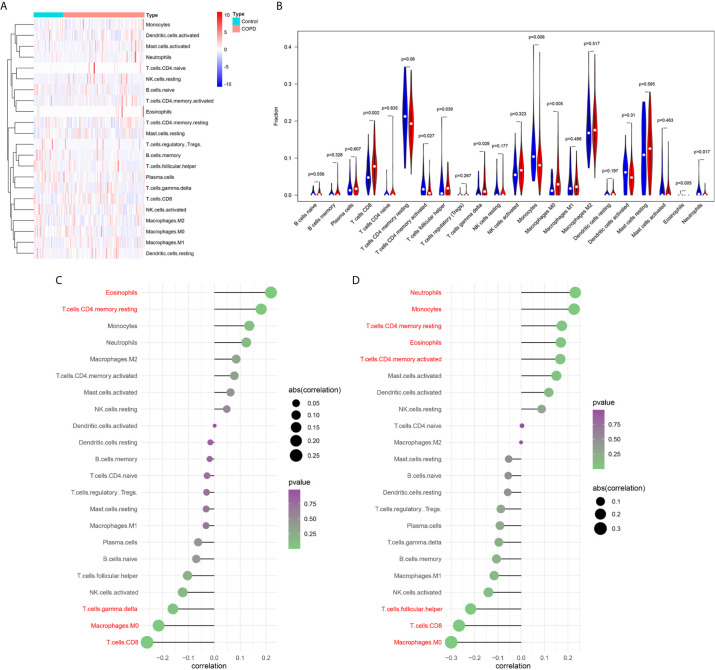
Analysis of immune landscape associated with COPD. Heatmap **(A)** and violin plot **(B)** showing the distribution of 22 types of immune cells in normal smoker and COPD patients in GSE76925; **(C)** The relationship between FVC% predicted and immune cell infiltration level; red: statistically significant (P < 0.05); **(D)** The relationship between FEV1/FVC and immune cell infiltration level; red: statistically significant (P < 0.05).

Functional enrichment analysis showed that immune related pathways were enriched in the COPD group compared with the normal smokers’ group. To explore the differential immune landscape in COPD patients and normal smokers, lung tissue microarray data from the GSE76925 dataset were analyzed. CIBERSORTx was used to estimate the fraction of 22 types of immune cells among the GSE76925 samples. CIBERSORTx is a website tool that enables evaluation of the relative proportion of immune cells in tissues *via* a deconvolution algorithm. The distribution of 22 types of immune cells in GSE76925 samples is shown in **Figure 2A**. The immune landscape results showed that T cells CD8, T cells follicular helper, T cells gamma delta, and macrophages M0 were upregulated, whereas T cells CD4 memory activated, monocytes, and eosinophils were downregulated in the lung tissues of COPD patients (**Figure 2B**). Next, we analyzed the relationship between immune infiltration and clinical features. As shown in **Figure 2C**, the infiltration level of neutrophils (r = 0.231, P = 0.004), monocytes (r = 0.226, P = 0.005), T cells CD4 memory resting (r = 0.174, P = 0.032), eosinophils (r = 0.170, P = 0.037), and T cells CD4 memory activated (r = 0.168, P = 0.039) were positively correlated with FEV1/FVC; the infiltration level of T cells follicular helper (r = -0.217, P = 0.007), T cells CD8 (r = -0.267, P < 0.001) and macrophages M0 (r = -0.300, P < 0.001) were negatively correlated with FEV1/FVC. Moreover, the infiltration levels of eosinophils (r = 0.219, P = 0.007) and T cells CD4 memory resting (r = 0.181, P = 0.026) were positively correlated with FVC% predicted; the infiltration levels of T cells gamma delta (r = -0.160, P = 0.049), macrophages M0 (r = -0.215, P = 0.008), and T cells CD8 (r = -0.260, P = 0.001) were negatively correlated with FVC1% predicted; the infiltration levels of T cells gamma delta (r = -0.160, P = 0.049), macrophages M0 (r = -0.215, P = 0.008), and T cells CD8 (r = -0.260, P = 0.001) were negatively correlated with FVC1% predicted (**Figure 2D**).

### Identification of Key Modules *via* WGCNA

**Figure 3:**
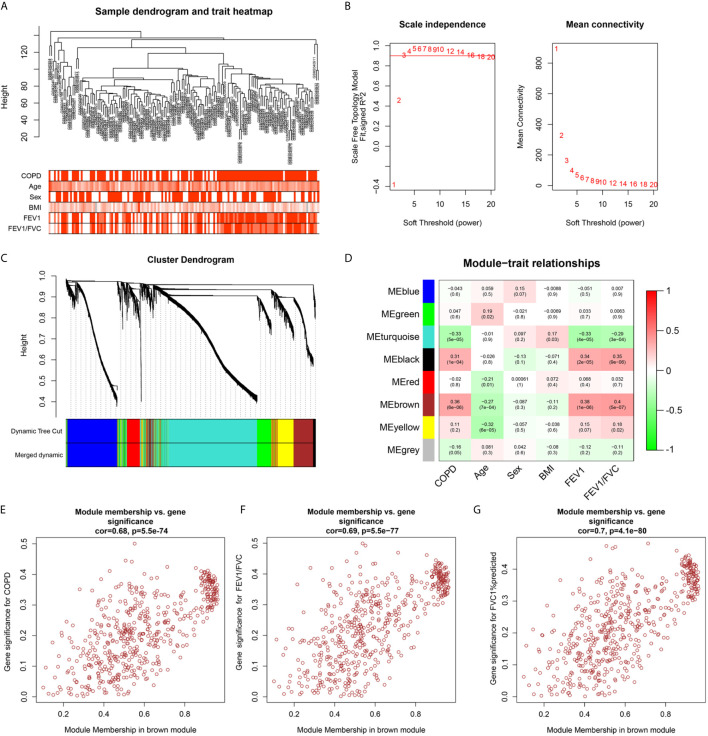
Weighted co-expression network analysis. **(A)** Sample dendrogram and trait heat map; **(B)** Analysis of the scale-free fit index (left) and the mean connectivity (right) for various soft-thresholding powers; **(C)** Clustering dendrograms of genes based on a dissimilarity measure (1-TOM); **(D)** Module-trait associations were evaluated by correlations between module eigengenes and sample traits. **(E–G)** Scatterplot of Gene Significance for COPD, FEV1/FVC, FEV1% predicted vs. Module Membership in brown module.

To identify the key genes related to the clinical features of COPD patients, co-expression network analysis was performed *via* WGCNA using the GSE76925 dataset. Clinical features (age, sex, BMI, FEV1/FVC, and FVC1% predicted) were obtained from the GSE76925 dataset. The parameters were established by setting the soft-threshold power to 3 (scale free R^2^ = 0.906) and the height was set to 0.25. In this study, 8 modules were identified (**Figures 3A–C**). The association between the modules and clinical features was measured by the correlation between module eigengene (ME) values and clinical features. Data were visualized by heatmap profiles. The results in **Figures 3D–G** showed that the brown module was the most closely corrected with COPD (Pearson co-efficient = 0.36, P = 6E-06), FEV1/FVC (Pearson co-efficient = 0.38, P = 1E-06) and FVC1%predicted (Pearson co-efficient = 0.4, P = 5E-07).

### Selection and Validation of Hub Genes

**Figure 4:**
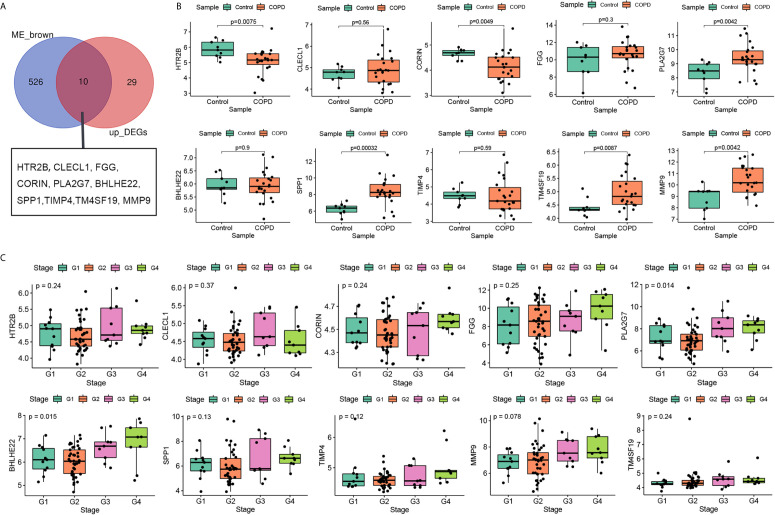
Selection and validation of hub gene. **(A)** Venn diagrams to indicate 10 shared genes from brown module and DEGs; **(B)** Validation of hub genes in the dataset GSE38974; **(C)** Validation of hub genes in the dataset GSE69818.

Previous results suggest that the expression level of genes in brown module increase with the pulmonary function decreases. Compared with normal subject, the pulmonary function of patients with COPD was decline, genes in brown module may up-regulated in lung tissue of patients with COPD. Therefore, to screen stable and robust hub genes accurately, ten commonly changed genes shared by the brown module and upregulated DEGs were selected (**Figure 4A**). These included HTR2B, CLECL1, FGG, CORIN, PLA2G7, BHLHE22, SPP1, TIMP4, TM4SF19, and MMP9. The expression levels of these ten genes were first validated in GSE38974 (nine smokers and 26 smokers with COPD). As shown in **Figure 4B**, the expression levels of HTR2B (P = 0.0075) and CORIN (P = 0.0049) were significantly lower in COPD lung tissues than in the smoker controls; the expression levels of PLA2G7 (P = 0.0042), SPP1 (P = 0.00032), TM4SF19 (P = 0.0087), and MMP9 (P = 0.0042) were significantly higher in COPD lung tissues than in the smoker controls. Next, the relationship between these ten genes and the GOLD stage was verified in GSE69818 (11 patients with GOLD stage 1, 41 patients with GOLD stage 2, nine patients with GOLD stage 3, and nine patients with GOLD stage 4). As shown in **Figure 4C**, the expression levels of PLA2G7 (P = 0.014) and BHLHE22 (P = 0.015) increased significantly with advanced GOLD stage. PLA2G7 was selected for subsequent analyses because it showed significant differences in two independent datasets.

### Functional Analysis and Validation of *PLA2G7*

**Figure 5:**
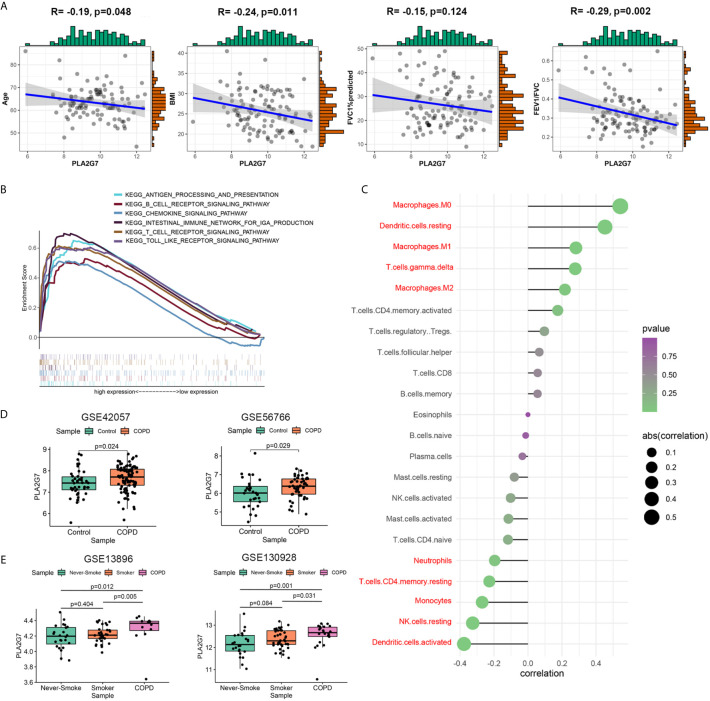
Function analysis and validation of PLA2G7. **(A)** The relationship between PLA2G7 and the clinical characteristics (age, BMI, FVC%predicted and FEV1/FVC) of COPD patients; **(B)** GSEA results showed that several immune-related pathways were significantly associated with PLA2G7; **(C)** Relationship between PLA2G7 expression and immune cell infiltration level; red: statistically significant (P < 0.05); **(D)** PLA2G7 expression was increased in blood of COPD patients based on GSE42057 (left) and GSE56766 (right); **(E)** PLA2G7 expression were up-regulated in alveolar macrophages of COPD samples based on GSE130928 (left) and GSE13896 (right).

The relationship between PLA2G7 and the clinical characteristics of COPD patients was analyzed. As shown in **Figure 5A**, age (r = -0.19, P = 0.048), BMI (r = -0.24, P = 0.011), and FEV1/FVC (r = -0.29, P = 0.002) were negatively correlated with PLA2G7 expression. To elucidate the potential regulatory mechanism of PLA2G7 in COPD, the GSE76925 dataset was used for GSEA analysis. COPD samples were divided into groups according to the median expression level of PLA2G7 (**Table S4**). As shown in **Figure 5B**, immune-related pathways “ANTIGEN PROCESSING AND PRESENTATION”, “B CELL RECEPTOR SIGNALING PATHWAY”, “CHEMOKINE SIGNALING PATHWAY”, “INTESTINAL IMMUNE NETWORK FOR IGA PRODUCTION”, “T CELL RECEPTOR SIGNALING PATHWAY” and “TOLL LIKE RECEPTOR SIGNALING PATHWAY” were enriched in the PLA2G7-high group. Therefore, we analyzed the relationship between immune infiltration and PLA2G7 expression. As shown in **Figure 5C**, the infiltration levels of macrophages M0 (r = 0.543, P < 0.001), dendritic cells resting (r = 0.452, P < 0.001), macrophages M1 (r = 0.282, P = 0.003), T cells gamma delta (r = 0.277, P = 0.003), and macrophages M2 (r= 0.217, P = 0.022) were positively correlated with PLA2G7 expression; the infiltration levels of neutrophils (r = -0.196, P = 0.039), T cells CD4 memory resting (r = -0.227, P = 0.017), Monocytes (r = -0.270, P = 0.004), NK cells resting (r = -0.325, P < 0.001), and dendritic cells activated (r = -0.376, P < 0.001) were negatively correlated with PLA2G7 expression.

To investigate whether PLA2G7 is differentially expressed in other tissues relevant to COPD, we analyzed a series of datasets. As shown in **Figure 5D**, PLA2G7 expression was significantly higher in the blood of COPD patients than in that of non-COPD controls (including 94 patients with COPD and 42 non-COPD controls from GSE42057 and 49 patients with COPD and 29 non-COPD controls from GSE56766). Given the significant correlation between PLA2G7 expression level and macrophages, we analyzed the differences in PLA2G7 expression in alveolar macrophages from bronchoalveolar lavage fluid (BALF). As shown in **Figure 5E**, there was no significant difference in the expression of PLA2G7 between never-smokers and normal smokers. Also, the expression levels of PLA2G7 in never-smokers and normal smokers were significantly lower than that of patients with COPD (including 22 patients with COPD, 24 never-smokers, and 42 smokers from GSE130928; and 12 patients with COPD, 24 never-smokers, and 34 smokers from GSE13896). These results showed that PLA2G7 expression was higher in different body fluid specimens from COPD patients than in those from normal controls, indicating that PLA2G7 may function in immune regulation by regulating macrophages.

### Validation of the PLA2G7 Encoded Protein Lp-PLA2 in Clinical Samples

**Figure 6:**
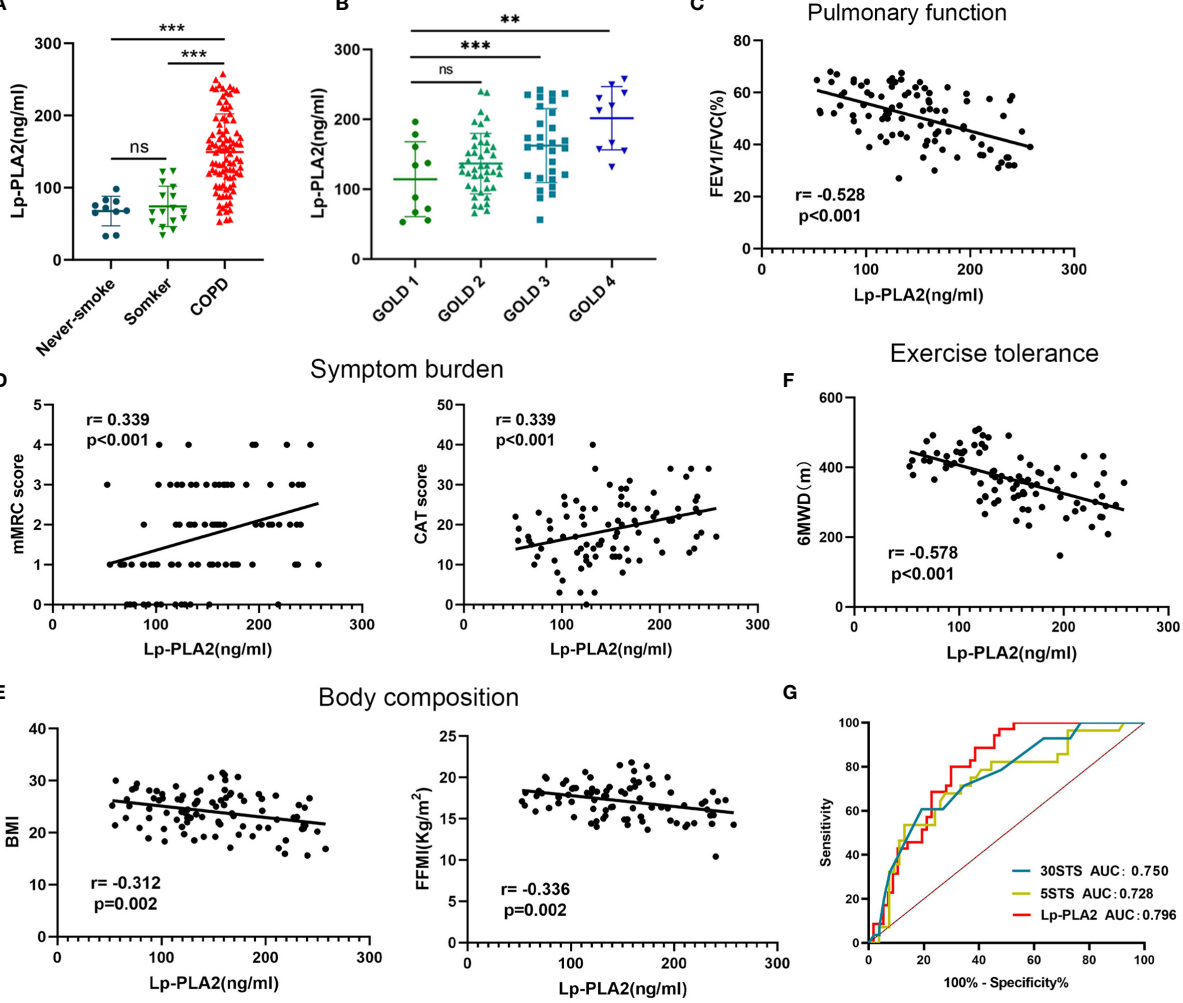
The clinical value of Lp-PLA2 level for COPD patients. **(A)** Lp-PLA2 level in serum tended to be higher in COPD patients than healthy smokers; **(B)** Lp-PLA2 level increase along with GOLD stage; **(C)** Lp-PLA2 level was negative correlate with FEV1/FVC (r=-0.528, p<0.001); **(D)** Lp-PLA2 level was positive correlate with mMRC score (r=0.339, p<0.001) (left) and CAT score (r=0.339, p<0.001) (right); **(E)** Lp-PLA2 level was negative correlate with BMI (r=-0.312, p=0.002) (left) and FFMI (r=-0.336, p=0.002) (right); **(F)** Lp-PLA2 level was negative correlate with 6MWD (r=-0.578, p=0.002); **(G)** ROC curve analysis of the Lp-PLA2 level, the 5STS score and the 30STS score for predicting 6MWD <350 m. ns, not statistically significant, **P < 0.01 and ***P < 0.001.

To verify the clinical application potential of the PLA2G7 gene, the levels of proteins encoded by the PLA2G7 gene were detected using ELISA in clinical samples. Lp-PLA2, which is encoded by the PLA2G7 gene, is a plasma enzyme bound to lipoproteins. There was no significant difference in the serum concentration of Lp-PLA2 between never-smokers and normal smokers. Also, the serum concentration of Lp-PLA2 in never-smokers and normal smokers were significantly lower than that of patients with COPD (**Figure 6A**). In addition, the expression of Lp-PLA2 increased in correlation with Gold stage (**Figure 6B**). Next, we analyzed the relationship between the expression of Lp-PLA2 and the clinical characteristics of COPD patients. Analysis of the relationship between Lp-PLA2 level and pulmonary function showed that Lp-PLA2 level was negatively correlated with FEV1/FVC (r = -0.528, P < 0.001) (**Figure 6C**).

GOLD states that in addition to the assessment of lung function, a comprehensive assessment of the clinical symptoms, acute exacerbations, and comorbidities of COPD is required. The CAT and the mMRC are widely used to assess the clinical symptoms of COPD patients (15). We analyzed the relationship between Lp-PLA2 levels and the CAT and mMRC scores. As shown in **Figure 6D**, Lp-PLA2 levels were positively correlated with the mMRC score (r = 0.339, P < 0.001) and CAT score (r = 0.339, P < 0.001).

Malnutrition has negative effects on exercise and muscle function, and on lung function, as well as increasing exacerbations and mortality (16). Body mass index (BMI) and fat-free mass index (FFMI) are used to assess nutritional status and are decreased in COPD patients (17). As shown in **Figure 6E**, Lp-PLA2 levels were negatively correlated with BMI (r = -0.312, P = 0.002) and FFMI (r = -0.336, P = 0.002).

Collectively, these findings suggested that Lp-PLA2 increased significantly in correlation with disease progression and is an important biomarker in COPD patients.

### Lp-PLA2 Level Effectively Evaluates Exercise Tolerance

Reduced exercise tolerance is one of the main clinical features of COPD. It increases the frequency of acute exacerbations and all-cause mortality, leading to a poor prognosis (4). The 6-min walk distance (6MWD) assesses the exercise tolerance of COPD patients (5). As shown in **Figure 6F**, Lp-PLA2 levels were negatively correlated with 6MWD (r = -0.578, P = 0.002).

Because finding an appropriate site is difficult (a 30 m flat course is required, and the layout of the track may influence the performance), the 6MWT is not common in primary medical institutions. We found that Lp-PLA2 level is highly correlated with 6MWD. Therefore, we explored whether Lp-PLA2 level can predict a poor 6MWD. The sit-to-stand test (STST) is widely used to indirectly evaluate exercise tolerance (18). Therefore, we compared the efficacy of the STST and Lp-PLA2 levels for predicting a poor 6MWD. A total of 35 patients (38.04%) had a 6MWD <350 m, with the remaining 57 patients (61.96%) demonstrating 6MWD >350 m. As shown in **Figure 6G**, the AUC of the 5STS score predicting a poor 6MWD (6MWD <350 m) was 0.728, the AUC of the 30STS score was 0.750, and the AUC of the Lp-PLA2 level was 0.796. The cutoff values of Lp-PLA2 level, 5STS, and 30STS scores were 133.7 ng/mL, 23.5, and 6.42, respectively. The sensitivity and specificity for predicting a poor 6MWD based on the cutoff value of the Lp-PLA2 level were 88.57% and 61.40%, respectively. The sensitivity and specificity for predicting a poor 6MWD according to the cutoff value of the 5STS score were 71.43% and 65.38%, respectively. The sensitivity and specificity for predicting a poor 6MWD based on the cutoff value of the 30STS score were 82.14% and 55.56%, respectively. These results suggested that the predictive power of Lp-PLA2 level is higher than that of STST modes, suggesting its potential for use in research and clinical practice.

### Construction of a Nomogram to Predict Impaired Exercise Tolerance

**Figure 7:**
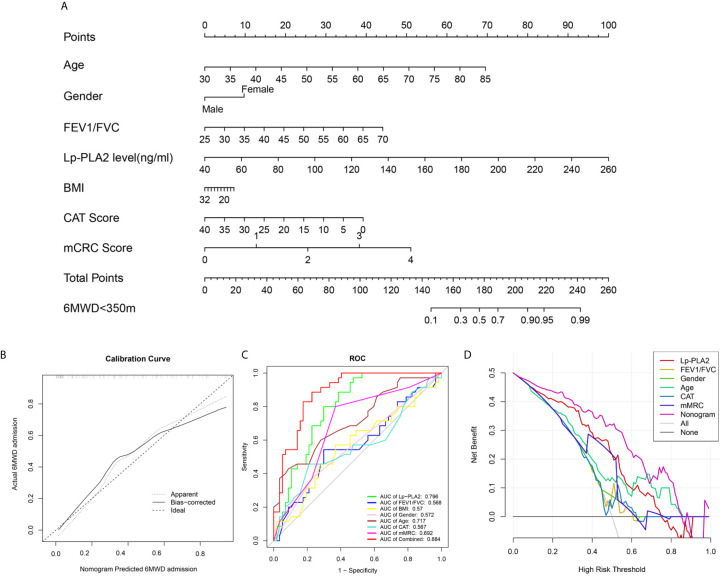
Construction of nomogram model. **(A)** Nomogram predicting 6MWD<350m for COPD patients; **(B)** Calibration curves for nomogram predicted 6MWD<350m for COPD patients; **(C)** ROC curve analysis show highest AUC value was seen for the nomogram model; **(D)** Decision curve analysis (DCA) shows the net benefit in 6MWD<350m predictions was the highest in nomogram model.

We combined the traditional clinical features of age, grade, FEV1/FVC, BMI, CAT score, and mMRC score with Lp-PLA2 level to construct a nomogram model to predict impaired exercise tolerance (6MWD <350 m) in COPD patients (**Figure 7A**). Calibration plots were used to visualize the performances of the nomograms. The calibration plot confirmed the performance of our model (**Figure 7B**). To demonstrate the clinical advantages of the nomogram model, we compared the ROC curves of the single variables against the nomogram curve. The nomogram model had the highest AUC value (**Figure 7C**). Finally, decision curve analysis (DCA) was used to confirm the findings. Compared with a single clinical variable, the combined nomogram model showed the highest efficacy for 6MWD <350 m predictions (**Figure 7D**). These methods confirmed the clinical utility of our nomogram model.

## Discussion

In this study, analysis of DEGs and WGCNA were combined to identify potential biomarkers related to the severity of COPD. PLA2G7 was selected for further analysis based on validation in independent datasets. We found that PLA2G7 levels were significantly correlated with BMI and pulmonary function and were increased in the blood and alveolar macrophages of COPD patients. Mechanistically, PLA2G7 may function *via* several immune-related pathways and macrophages. The present study is the first report demonstrating the clinical value of PLA2G7 in COPD and suggesting its potential as a biomarker for COPD.

Several studies have identified biomarkers of COPD based on gene expression profiles from public databases. Differentially expressed genes (DEGs) analysis between patients with COPD and control subjects was firstly used by researchers to explore markers of COPD. Several studies (19–22) explore a series of biomarkers *via* DEGs analysis based on single public database from GEO database. In additional, DEGs analysis based on multiple gene expression dataset was also used to explore potential biomarker (23). However, confounding factors reduces both the sensitivity and the specificity of the DEGs as biomarkers for COPD. Therefore, it is important to understand the relationship between DEGs and clinical phenotype (24). WGCNA, as widely used bioinformatics method, focused on the relationship between co‐expression modules and clinical traits (25). Several COPD biomarkers studies (26–28) explore gene modules correlate with clinical features (emphysema severity, lung function, etc.) of patients with COPD. Our study applies DEGs analysis combined with WGCNA to explore novel hub genes related to clinical features of patients with COPD. And our study is the first to verify key genes in both different public datasets and independent clinical cohort and to analyses the predictive efficiency of hub gene encoding protein (Lp-PLA2) level in exercise tolerance. Importantly, a nomogram model was constructed combine traditional clinical features and Lp-PLA2 for improve predictive power or specificity.

Our study analysis the differences of immune infiltrating cells between COPD tissue and normal lung tissue. In additional, we analysis the relationship between immune infiltrating cells and pulmonary function of patients. First, our result indicates that the imbalance of T lymphocyte subsets (CD8^+^ T cells, T cells follicular helper, T cells gamma delta and T cells CD4 memory activated) in lung tissue was significant correlate with poor pulmonary function. CD8^+^ T cells is a cytotoxic T lymphocyte that secretes various cytokines (granzymes, perforin, etc.) to participate in the immune function (29–31). Several studies (32–35) have found the elevated expression of the CD8^+^ T cells in lung tissue, bronchoalveolar lavage fluid or induced sputum of patients with COPD. Increased expression of cytotoxic mediators by the already increased number of CD8^+^ T cells contributes to lysis of structural cells in the lung, leading emphysema (36). The findings in our study are consistent with previous reports as we found that the infiltration level of CD8^+^ T cells was up-regulated in lung tissue of patients with COPD, and relate to poor pulmonary function. Follicular T helper (Tfh) cells was a subset of CD4^+^ T cells, play important roles in facilitating B‐cell differentiation and tertiary lymphoid organs (TLOs) (37). Recent study indicates that lung dendritic cells could induce Tfh cells, working together in TLO formation (38). TLO plays a crucial role in COPD progression (39, 40). Our result confirms that the infiltration level of Tfh cells were negatively correlated with pulmonary function in patients with COPD. T cells gamma delta (γδT cells) are an unconventional subset of T lymphocytes that play an indispensable role in the homeostasis of the immune system (41, 42). Pulmonary intraepithelial γδT-cells could repair lung tissue and protection of the lung against environmental stimuli (43, 44). Previous studies (45, 46) indicate that the numbers of γδT-cells in induced sputum and in bronchoalveolar lavage of COPD patients was significantly lower than healthy subjects. And, the quantity of γδT-cells in COPD group was negatively correlated with forced expiratory volume in 1 s (45). Our result suggests that γδT-cells was increased in lung tissue of patients with COPD, may function as a protector. T cells CD4 memory activated (CD4^+^ TM) in lung tissue, serving as ‘first line’ of defense at barrier sites, recruiting CD4^+^ and CD8^+^ T‐cells, and facilitate rapid immune defense (47, 48). In this study, we found CD4^+^ TM cells were down-regulated in lung tissue of patients with COPD, which may lead more susceptible to infection by bacteria or viruses.

We also found several innate immune cells were differential expressed between lung tissue with COPD and normal lung tissue. First, our result indicate that macrophages M0 were upregulated and monocytes were downregulated in lung tissue of COPD patients. Macrophages cause lung destruction through the release of oxygen radicals and proteolytic enzymes (49, 50). Several studies have shown that eosinophils and neutrophils are involved in chronic inflammation and tissue damage through the secretion of inflammatory mediators (51, 52). In this study, we also found eosinophils and neutrophils were abnormal expressed in lung tissue with COPD.

To explore the clinical application value of PLA2G7, we recruited COPD patients and healthy smokers, and detected the expression levels of the PLA2G7-encoded protein Lp-PLA2. Lp-PLA2 is mainly produced by macrophages and activated platelets (53, 54). Lp-PLA2 functions as an inflammatory biomarker for cardiovascular and cerebrovascular diseases (55, 56). Systemic inflammation in COPD is a risk factor for reduced exercise tolerance (57, 58). In this study, Lp-PLA2 was upregulated in COPD patients compared with never-smokers and normal smokers, and increased along with GOLD stage. It was significantly correlated with clinical symptoms, nutritional status, and exercise tolerance in COPD patients. These results suggest that Lp-PLA2 is a potential biomarker for COPD. Because Lp-PLA2 level was highly correlated with 6MWD, we explored the ability of Lp-PLA2 level to predict a poor 6MWD. The 6MWT is a measure of exercise capacity (5). However, the 6MWT requires a suitable venue and professional training, and it is thus difficult to use in the busy clinical setting. Developing a screening method that is highly accurate, simple, and can be performed in any medical facility is important. Studies from our team and other group shows that STST is an effective method to indirectly assess exercise tolerance (14, 59). In this study, we compared the ability of the STST modes and Lp-PLA2 levels for predicting a poor 6MWD. Lp-PLA2 level had the highest AUC value (0.796), highest sensitivity (88.57%), and moderate specificity (61.40%), indicating that it is a useful predictor of a poor 6MWD compared with the 5STS and the 30STS. A nomogram model constructed by combining traditional clinical features and Lp-PLA2 level further improved the predictive ability (AUC: 0.884). The present results suggest that serum Lp-PLA2 levels and the nomogram model are simple and accurate methods to predict exercise tolerance in patients with COPD.

The present study had several limitations. First, we only detected the expression of Lp-PLA2 in the serum, and its expression level in sputum and alveolar lavage fluid remains unknown. These two kinds of samples are more closely related to airway inflammation. Secondary, the clinical application of Lp-PLA2 as a potential biomarker needs to be further assessed and subjected to external verification. These data will be provided in a future study. And the datasets used in this study are all microarray data. However, we failure to apply RNA-seq data in our study, which is an advanced technology. We hope to collect more RNA-seq data in future study. Finally, due to the limitation of the volume of clinical samples, we are unable to verify all the up-regulated genes (including SPP1, TM4SF19 and MMP9). It appears that a single biomarker would lack enough predictive power. Multiple biomarkers should be included to build a predictive model to improve predictive power. Unable to incorporate more hub genes is one of the limitations of our study. In future research, we will try to use new statistical methods (such as, random forest method, least absolute shrinkage and selection operator regression) to build a new predictive model to improve predictive power or specificity.

In conclusion, our results strongly suggested Lp-PLA2 is a promising biomarker for COPD patients, and it is suitable for predicting poor exercise tolerance in clinical practice.

## Data Availability Statement

Publicly available datasets were analyzed in this study. This data can be found here: GEO database, accession number: GSE76925.

## Ethics Statement

The studies involving human participants were reviewed and approved by the Research Ethics Committee of China Medical University. The patients/participants provided their written informed consent to participate in this study.

## Author Contributions

MD: Investigation, Data curation, Writing – original draft. YY: Investigation, Data curation. QZ: Methodology. XZ: Writing - review and editing. GH: Conceptualization, Writing - review and editing, Project administration. All authors contributed to the article and approved the submitted version.

## Funding

This research was supported by the Non-profit Central Research Institute Fund of Chinese Academy of Medical Sciences (No. 2020-PT320-001), and National Natural Science Foundation of China (No. 81900040, 81700041).

## Conflict of Interest

The authors declare that the research was conducted in the absence of any commercial or financial relationships that could be construed as a potential conflict of interest.

## Supplementary Material

Supplementary Table 1 GO analysis (biological processes) of the 352 DEGs

Supplementary Table 2 Pathways were enriched in the smokers group

Supplementary Table 3 Pathways were enriched in the COPD group

Supplementary Table 4 Pathways were enriched in the PLA2G7-high group.
